# *In Vitro* and *In Vivo* Activity of Ribavirin against Andes Virus Infection

**DOI:** 10.1371/journal.pone.0023560

**Published:** 2011-08-10

**Authors:** David Safronetz, Elaine Haddock, Friederike Feldmann, Hideki Ebihara, Heinz Feldmann

**Affiliations:** 1 Laboratory of Virology, Division of Intramural Research, National Institute of Allergy and Infectious Diseases, National Institutes of Health, Hamilton, Montana, United States of America; 2 Office of Operations and Management, Division of Intramural Research, National Institute of Allergy and Infectious Diseases, National Institutes of Health, Hamilton, Montana, United States of America; 3 Department of Medical Microbiology, University of Manitoba, Winnipeg, Manitoba, Canada; Tulane School of Public Health and Tropical Medicine, United States of America

## Abstract

Pathogenic hantaviruses are a closely related group of rodent-borne viruses which are responsible for two distinct diseases in humans, hemorrhagic fever with renal syndrome and hantavirus pulmonary syndrome (HPS, otherwise known as hantavirus cardiopulmonary syndrome, HCPS). The antiviral effect of ribavirin against Old World hantaviruses, most notably Hantaan virus, is well documented; however, only a few studies have addressed its inhibitory effect on New World hantaviruses. In the present study, we demonstrate that ribavirin is highly active against Andes virus (ANDV), an important etiological agent of HPS, both *in vitro* and *in vivo* using a lethal hamster model of HPS. Treatment of ANDV infected Vero E6 cells with ribavirin resulted in dose-dependent reductions in viral RNA and protein as well as virus yields with a half maximal inhibitory concentration between 5 and 12.5 µg ml^−1^. In hamsters, treatment with as little as 5 mg kg^−1^ day^−1^ was 100% effective at preventing lethal HPS disease when therapy was administered by intraperitoneal injection from day 1 through day 10 post-infection. Significant reductions were observed in ANDV RNA and antigen positive cells in lung and liver tissues. Ribavirin remained completely protective when administered by intraperitoneal injections up to three days post-infection. In addition, we show that daily oral ribavirin therapy initiated 1 day post-infection and continuing for ten days is also protective against lethal ANDV disease, even at doses of 5 mg kg^−1^ day^−1^. Our results suggest ribavirin treatment is beneficial for postexposure prophylaxis against HPS-causing hantaviruses and should be considered in scenarios where exposure to the virus is probable. The similarities between the results obtained in this study and those from previous clinical evaluations of ribavirin against HPS, further validate the hamster model of lethal HPS and demonstrate its usefulness in screening antiviral agents against this disease.

## Introduction

Hantaviruses (family *Bunyaviridae*, genus *Hantavirus*) are a global threat to public health and are associated with two diseases in humans, hemorrhagic fever with renal syndrome (HFRS) and the more recently recognized hantavirus pulmonary syndrome (HPS), also known as hantavirus cardiopulmonary syndrome (HCPS) to highlight the importance of cardiogenic shock in severe disease. Currently, the International Committee on the Taxonomy of Viruses recognizes at least 22 unique species of hantaviruses, half of which are pathogenic to humans [1]. Mortality rates associated with confirmed hantavirus cases range up to 10% with HFRS, and 40–50% with HPS [2]. In nature, human pathogenic hantaviruses are maintained in rodent hosts which, once infected, are believed to carry and shed the virus asymptomatically for life. Humans typically become infected by inhalation of virus contaminated rodent excreta/secreta, although for Andes virus (ANDV), an important etiological agent of HPS in South America, human to human transmission has been documented [3], [4].

Despite significant morbidity and mortality associated with these viruses, currently no FDA approved vaccine exists for the prevention of HFRS or HPS and therapeutic treatment options are limited [2], [5]. Ribavirin (1-β-D-ribofuranosyl-1, 2, 4-triazole-3-carboxamide) is a broad spectrum synthetic guanosine analogue with virustatic activity against a number of DNA and RNA viruses, including bunyaviruses [6]–[8]. Ribavirin has been shown to be effective at reducing Hantaan virus replication *in vitro* [7], [9]–[11], and *in vivo* using suckling mice [9], [12]. In addition, a two year prospective clinical trial conducted in China demonstrated that intravenous ribavirin therapy had a statistically significant positive effect, in HFRS patients, with reduced morbidity and mortality observed in the treatment group when compared to the placebo control group [13]. These findings are supported by a recent study conducted in Korea which found treatment with intravenous ribavirin resulted in decreased renal complications compared to a non-ribavirin treated cohort of confirmed HFRS cases [14].

Although the antiviral activity of ribavirin against Old World, HFRS-causing hantaviruses is documented, its efficacy against HPS-causing New World hantaviruses is less clear [2], [15]. Medina and colleagues showed that ribavirin inhibited Sin Nombre virus (SNV) infection *in vitro* and demonstrated that pre-treatment of deer mice, followed by daily therapy with 100 mg ribavirin kg^−1^ reduced SNV infection as measured by seroconversion, viral RNA synthesis and immunohistochemistry (IHC), while treatment with 50 mg kg^−1^ or less had a reduced inhibitory effect [16]. Two attempts have been made to assess the value of ribavirin therapy in patients with HPS in North America and although hampered by low enrollment rates, the results of these studies showed no effect of ribavirin therapy when treatment was initiated during the symptomatic phase of infection [17], [18]. The purpose of this study was to evaluate the antiviral activity of ribavirin against ANDV, both *in vitro* and *in vivo* using a lethal hamster model of HPS [19], [20]. Our results show that ribavirin has an inhibitory effect on ANDV replication and post-exposure treatment of hamsters with as little as 5 mg ribavirin kg^−1^ day^−1^ was effective at preventing lethal HPS disease. Further, we show ribavirin therapy remained 100% effective when administered three days post-infection (p.i.). The similarities of the results obtained here and those from the clinical trials aimed at assessing the antiviral effect of ribavirin against HPS caused by SNV further validate the hamster model of HPS.

## Materials and Methods

### Ethics statement

All animal experiments were approved by the Institutional Animal Care and Use Committee of the Rocky Mountain Laboratories (approval ID 2011-14), and performed following the guidelines of the Association for Assessment and Accreditation of Laboratory Animal Care, International (AAALAC) by certified staff in an AAALAC approved facility.

### *In vitro* experiments

ANDV, strain Chile 9717869 [21] was propagated and titered as previously described [22]. The half maximal inhibitory concentration (IC_50_) of ribavirin against ANDV was calculated essentially as previously described [16]. Briefly, nearly confluent Vero E6 cells (ATCC, Manassas VA) were infected with ANDV in 24-well plates for 1 hour at a multiplicity of infection (m.o.i.) of 0.01. After absorption, cells were washed and the inoculum was replaced with 0.5 ml of Dulbecco's modified Eagle's medium (DMEM) supplemented with 2% fetal bovine serum (FBS), antibiotics (penicillin [100 U ml−1] and streptomycin [100 µg ml−1]), L-glutamine (2 mM), and varying concentrations (0, 1, 2.5, 5, 12.5 25, 50 or 100 µg ml−1) of ribavirin. Supernatant (0.5 ml) was collected on days 1, 3, 5 and 7 p.i. and replaced with fresh media containing ribavirin, as above. Infectious ANDV titers were determined using a focus assay as previously described [16], [22].

The effect of ribavirin treatment on viral RNA and protein synthesis was also assessed *in vitro*. Nearly confluent monolayers of Vero E6 cells were infected with ANDV (m.o.i. 0.01) in 6-well dishes and treated in duplicate with ribavirin (0, 5, 25, 50 or 100 µg ml^−1^) as above. On days 1, 3 and 7 p.i., supernatant was collected and inactivated in lysis buffer AVL (Qiagen, Valencia CA). Infected cells were harvested by low speed centrifugation, divided in two fractions and inactivated with either lysis buffer RLT (Qiagen) or SDS loading buffer.

### Western blot

Samples were boiled for 5 minutes and proteins were separated by SDS-PAGE and electrophoretically transferred to a nitrocellulose membrane. The ANDV nucleoprotein was detected with chemiluminescence using a primary monoclonal antibody (1∶10000 dilution, clone 1A8F6, Austral Biologicals, San Ramon CA) and a peroxidase labeled anti-mouse secondary antibody (1∶10000 dilution, Jackson Immunoresearch laboratories, Inc. West Grove PA). An anti-beta actin primary monoclonal antibody was included as a loading control (1∶500 dilution, Santa Cruz biotechnology, Inc. Santa Cruz CA).

### *In vivo* experiments

Syrian hamsters (*Mesocricetus auratus*, female, aged 4–6 weeks, Harlan Laboratories Inc, Indianapolis IN) were inoculated with 100×LD_50_ of ANDV (representing an approximate challenge dose of 154 focus forming units, FFU) diluted in sterile DMEM by intraperitoneal (i.p.) injection. Unless otherwise indicated, ribavirin treatments were administered daily by i.p. injections of 100 µl of drug diluted to the indicated concentration in sterile PBS. The first experiment consisted of a dose-response study in which five groups of 12 hamsters were inoculated with ANDV and treated with ribavirin (100, 50, 25 or 5 mg kg^−1^ day^−1^ or PBS alone) beginning 1 day p.i., for 10 consecutive days. On days 6 and 8 p.i., three anesthetized hamsters per treatment group were exsanguinated by cardiac puncture and lung and liver samples were collected and inactivated in 10% neutral buffered formalin or lysis buffer RLT. Blood samples were inactivated in lysis buffer AVL. The remaining 6 animals per group were monitored for survival for 35 days p.i.

In follow-up experiments, the efficacy of abbreviated or delayed ribavirin therapy was assessed. In the first follow-up, three groups of six ANDV infected hamsters were treated daily with ribavirin (50 mg kg^−1^ day^−1^) beginning 1 day p.i. and terminating on days 3, 5 or 7 p.i. To evaluate the efficacy of delayed ribavirin treatments, three groups of six ANDV infected hamsters were treated with ribavirin (50 mg kg^−1^ day^−1^) for 10 days beginning on days 3, 5 or 7 p.i. For each treatment group in the two follow-up experiments, a control group of three hamsters was infected and treated with PBS alone according to the same therapeutic schedule.

In a final experiment, the protective efficacy of oral ribavirin therapy was assessed. Hamsters were infected with ANDV as outlined above and, beginning at day 1 p.i., treated with 100 µl of ribavirin at the indicated concentrations by oral gavage using a stainless steel 18 G ball-tipped feeding needle, for 10 consecutive days. Two groups of 6 hamsters received ribavirin at 5 or 50 mg kg^−1^ day^−1^ diluted in sterile PBS, and a control group of 3 hamsters received PBS alone. Chest radiographs were taken from representative hamsters of each treatment group using a portable digital radiography unit with a flat panel digital detector (TruDR, Sound-Eklin, Carlsbad CA) and veterinary specific software (VET-PACS, London, United Kingdom) at the time of euthanasia of the control treated hamsters.

All work with infected hamsters and potentially infectious materials derived from hamsters was conducted in a Biosafety Level 4 facility at the Rocky Mountain Laboratories. Sample inactivation and removal was performed according to standard operating protocols approved by the local Institutional Biosafety Committee.

### Histology and Immunohistochemistry (IHC)

Formalin fixed tissues were embedded in paraffin, processed according to standard procedures and stained with hematoxylin and eosin or tested for the presence of viral antigen by IHC using an ANDV nucleoprotein specific monoclonal antibody (1∶500 dilution, clone 1A8F6, Austral Biologicals). IHC results were confirmed using a polyclonal rabbit antiserum generated against the Sin Nombre virus nucleoprotein [16]. Slides were evaluated by a veterinary pathologist and IHC slides were numerically scored based on the percentage of immunoreactive cells as follows: 1 = 1–25%; 2 = 26–50%; 3 = 51–75%; 4 = 76–100%.

### ANDV specific quantitative RT-PCR

Total RNA was extracted from cells and solid tissue (approximately 30 mg pieces) using RNeasy mini kits and from cell culture supernatant and blood using QIAamp viral RNA kits (both from Qiagen). Viral RNA was quantified on a rotor-gene 6000 instrument (Corbett Life Science, Sydney Australia) using QuantiFast probe reagents (Qiagen) with a previously described real-time RT-PCR assay specific for the ANDV nucleoprotein coding region [22].

### Transcriptional profiling of host responses

Host responses, including interleukin (IL)-6 and IL-10, tumor necrosis factor (TNF) α, interferon (IFN) γ, myxovirus resistance protein (Mx) 2 and signal transducer and activator of transcription (STAT)-1 were monitored in RNA extracted from lung samples collected at day 8 p.i. from treated and control animals using Syrian hamster-specific, quantitative real-time RT-PCR assays, as previously described and using ribosomal protein L18 as an internal control [23]. Transcriptional up or down regulation was determined by comparing data from treated and untreated infected hamsters to that obtained from uninfected control hamsters that were injected with PBS alone according to the same schedule.

### Serology

Antibodies against the ANDV nucleoprotein are cross-reactive to the homologous SNV antigen [24], therefore, a recombinant SNV nucleoprotein-based ELISA was employed to assess seroconversion to ANDV in convalescent serum samples collected from surviving hamsters, as previously described [25].

### Statistical analysis

Statistical differences between treatment groups were examined using a one-way analysis of variance with Tukey-Kramer multiple comparison post-test. Survival rates were compared using Fisher's exact test.

## Results

### *In vitro* effect of ribavirin on ANDV replication and viral RNA and protein synthesis

**Figure 1:**
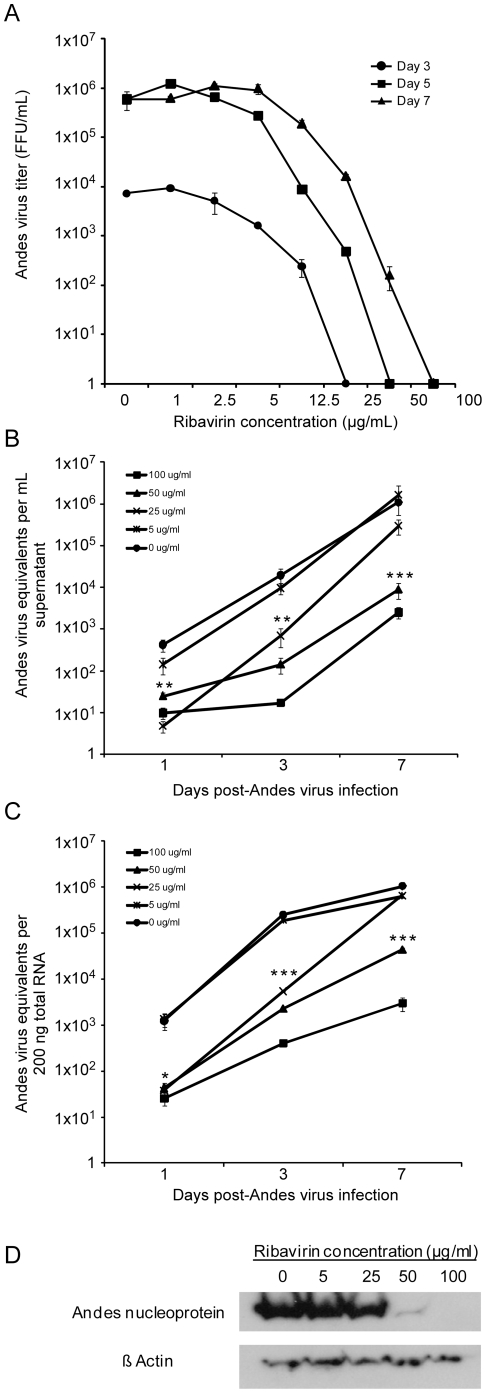
*In vitro* effect of ribavirin against Andes virus. Vero E6 cells were infected with Andes virus (m.o.i. = 0.01) and cultured in the presence of varying concentrations of ribavirin. (A) Supernatants collected at 3, 5 and 7 days post-infection were titered using a focus forming unit (FFU) assay. Day 1 supernatants were also collected, though titers were below the limit of detection of the focus assay. Data represent the average of three measurements. Error bars represent the standard error of the mean. (B–C) Andes virus RNA concentrations in supernatant (B) and Vero E6 cells (C) were determined using quantitative real-time RT-PCR targeting the nucleoprotein coding region. Error bars represent the standard error of the mean. (D) Andes virus nucleoprotein expression levels in infected Vero E6 cells cultured in the presence of ribavirin for 7 days post-infection. * p<0.05, ** p<0.01,*** p<0.0001.

Treatment of ANDV infected cells with ribavirin had a dose-dependent inhibitory effect on infectious virus yields and detection of viral RNA and proteins. The IC_50_ of ribavirin against ANDV was between 5 and 12.5 µg ml^−1^ as determined from infectious titers calculated in supernatants collected on days 3, 5 and 7 p.i. (Figure 1A). Day 1 p.i. supernatants were collected; however, infectious titers were below the limit of detection of the focus forming unit assay. Reductions of viral RNA in the cell culture supernatant of ANDV infected Vero cells treated with ribavirin were observed throughout the course of a 7 day infection, with statistically significant differences noted on days 1 (p = 0.0023) and 3 p.i. (p = 0.0073) at ribavirin concentrations of 25 µg ml^−1^ or greater and on day 7 p.i. at concentrations of 50 µg ml^−1^ or greater (p<0.0001, Figure 1B). Similar reductions in viral RNA were observed in cell lysates with statistically significant results noted on days 1 (p = 0.0161) and 3 p.i. (p<0.0001) in cells treated with ribavirin at concentrations of 25 µg ml^−1^ or greater and on day 7 p.i. at concentrations of 50 µg ml^−1^ or greater (p<0.0001, Figure 1C). Consistent with these findings, reductions in ANDV nucleoprotein synthesis was observed in infected cells in the 50 and 100 µg ml^−1^ treatment groups (Figure 1D).

### *In vivo* efficacy of ribavirin against lethal ANDV infection

**Figure 2:**
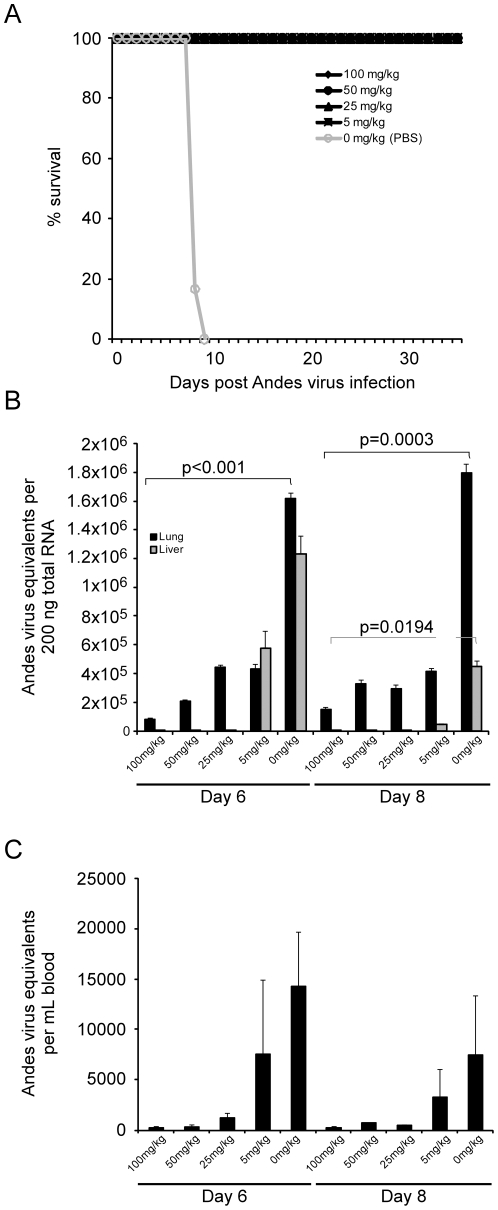
Effect of daily ribavirin therapy on lethal Andes virus infection in hamsters. Groups of 12 hamsters were infected with a lethal dose of Andes virus and treated by daily intraperitoneal injections of 0, 5, 25, 50 or 100 mg ribavirin kg^−1^ day^−1^ from day 1 through day 10 post-infection. For each group, 6 hamsters were monitored for survival (A), while 3 hamsters per group were euthanized on days 6 and 8 post-infection and samples collected for virological analysis (B–C). Andes virus RNA profiles were determined in lung and liver (B) and blood (C) samples collected from hamsters as noted above. The data represents the average values obtained from triplicate analysis of tissue samples collected from three hamsters per treatment group, per time point. Error bars represent the standard error of the mean.

Treatment of ANDV infected hamsters with ribavirin at concentrations of 100, 50, 25 or 5 mg kg^−1^ day^−1^ from day 1 through day 10 p.i. afforded 100% protection against lethal disease (Figure 2A). Animals treated with ribavirin at concentrations of 25 mg kg^−1^ day^−1^ or greater showed no overt signs of ANDV infection throughout the course of study, while 50% of hamsters treated with 5 mg kg^−1^ day^−1^ appeared to have mild signs of infection including lethargy and minimal breathing abnormalities between days 7 and 10 p.i. Control (PBS treated) hamsters began showing signs of illness including lethargy and hunched posture on days 7–8 p.i. Within 12–24 hours breathing abnormalities leading to severe respiratory distress were apparent in all control hamsters, with some also showing cyanosis and/or epistaxis. All control hamsters succumbed to ANDV infection between days 8 and 10 p.i. (approximately 24–48 hours after initial signs of illness). The survival rate associated with i.p. administration of ribavirin treatments at all concentrations was statistically significant (p = 0.0022).

Daily ribavirin therapy resulted in reduced viral load in tissue samples collected at days 6 and 8 p.i. (Figure 2B). Comparison of viral RNA levels in lung samples from ribavirin treated versus control hamsters revealed a statistically significant reduction in ANDV RNA at both the day 6 and 8 p.i. sampling points (p<0.0001 and p = 0.0003, respectively). Similar reductions in ANDV RNA in liver samples were also observed, most notably in samples collected at day 8 p.i. in the 100, 50 and 25 mg kg^−1^ day^−1^ treatment groups (p = 0.0194). A dose-dependent reduction in viral RNA in blood samples was noted, especially in hamsters receiving 25 mg ribavirin kg^−1^ day^−1^ or greater (Figure 2C).

**Figure 3:**
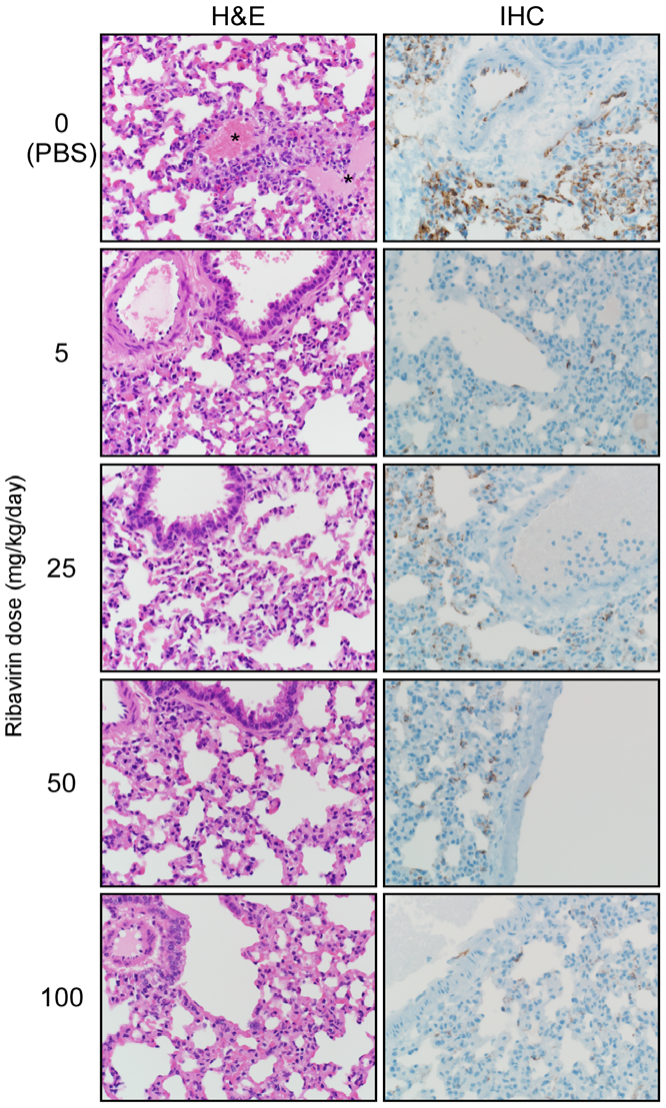
Histological analysis of lungs from Andes virus infected ribavirin treated and control hamsters. Hamsters were infected with a lethal dose of Andes virus and treated daily with ribavirin at the indicated concentrations from day 1 through day 10 post-infection. Shown are hematoxylin and eosin (H&E) and immunohistochemistry (IHC) stained sections of lungs collected at day 8 post-infection from treated and control hamsters. Histological abnormalities were only noted in control hamsters which demonstrated perivascular edema (see *). IHC with a monoclonal antibody (shown) and polyclonal sera (not shown) revealed drastic reductions in the detection of Andes virus nucleoprotein in all ribavirin treated hamsters compared with the diffuse staining noted in the lung endothelium of control (PBS) treated animals.

Immunohistochemical examination of lung and liver samples from control and treated animals revealed a drastic reduction in cells reactive for hantaviral antigen in hamsters treated with ribavirin. Lung sections from control, PBS treated hamsters demonstrated multifocal to coalescing immunopositivity in pulmonary alveolar and arteriolae endothelium, with up to 90% of tissue affected (severity score 4, Figure 3). Sections from hamsters treated with ribavirin also displayed multifocal immunopositivity in pulmonary endothelium, however on average less than 40% of the tissue was affected (severity score 1–2, Figure 3). In liver sections from control hamsters, mid-zonal hepatocytes were widely positive as were multifocal endothelial cells lining arterioles and hepatic sinusoids (score 2–3, data not shown). In contrast, liver sections from hamsters treated with ribavirin rarely demonstrated any positive hepatocytes or endothelial cells (score 0–1, data not shown). The pattern of staining in lung and liver sections was similar in samples collected at both 6 and 8 days p.i. Decreases in viral antigen were similar using both a monoclonal antibody and polyclonal sera suggesting the reduced antigen detection was not an artifact associated with potential mutations within antibody epitopes.

**Figure 4:**
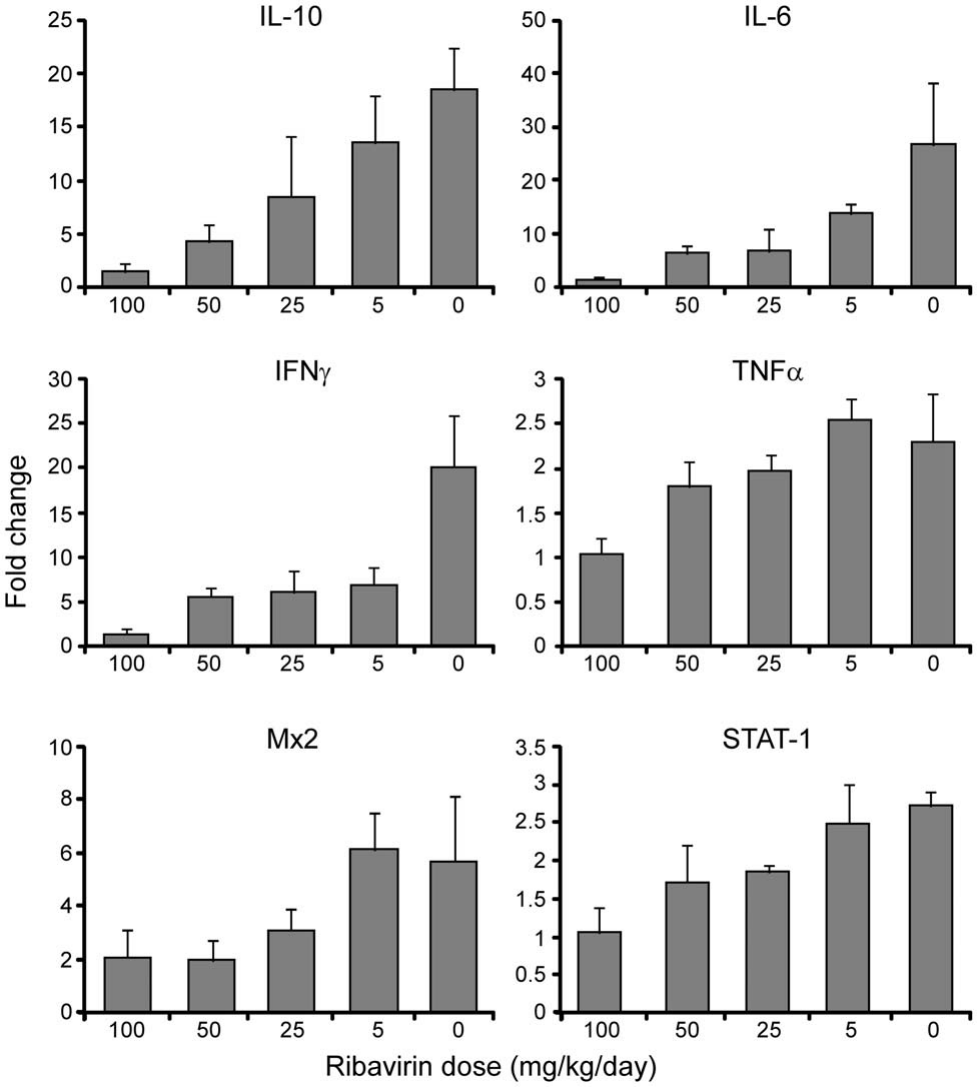
Analysis of host responses in ribavirin treated and control animals. Hamsters were infected with a lethal dose of Andes virus and treated daily with ribavirin at the indicated concentrations administered by intraperitoneal injections. Host responses to infection were monitored in lung samples collected at 8 days post-infection using recently developed, hamster specific, real-time RT-PCR assays. Shown are the average fold changes of triplicate analysis of samples collected from 3 hamsters per treatment group. Data is normalized to uninfected animals and error bars represent the standard error of the mean.

Consistent with the noted decreases in viral burden, disease progression was limited to the minor physical signs noted above in the lowest treatment group. Histological examination of lung sections collected 8 days p.i. demonstrated mild, multifocal perivascular edema in control hamsters, which was absent in ribavirin treated hamsters (Figure 3). Analysis of host immune responses found dose dependant decreases in transcriptional levels of Th1 and Th2 cytokine genes including IL-10, IL-6 and IFN γ, and to a lesser extent TNF α, in lung samples from ribavirin treated hamsters as compared to untreated infected hamsters (Figure 4). The innate markers Mx2 and STAT-1 were also not transcriptionally activated to the same extent as infected, PBS treated hamsters, most notably in the higher treatment groups (Figure 4).

### Effect of abbreviated or delayed ribavirin therapy on lethal ANDV disease in hamsters

**Figure 5:**
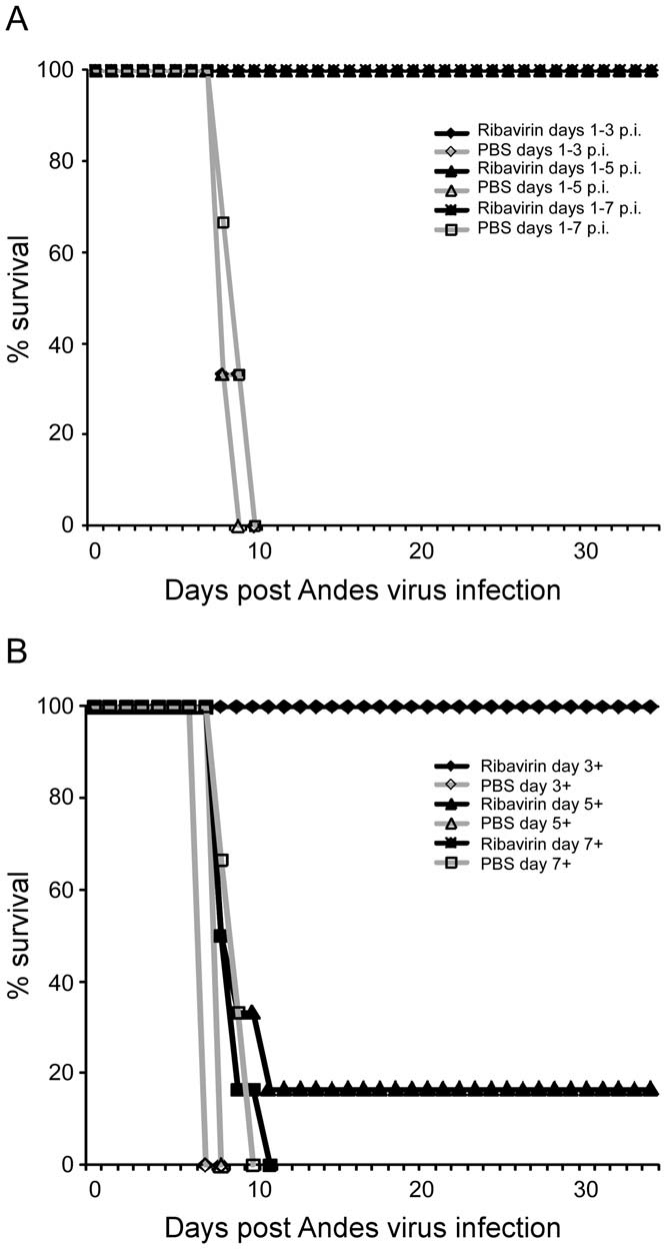
Effect of abbreviated or delayed ribavirin therapy on lethal Andes virus infection in hamsters. Hamsters were infected with a lethal dose of Andes virus as described in the materials and methods section and treated with ribavirin at 50 mg kg^−1^ day^−1^ as outlined below. (A) Effect of abbreviated ribavirin therapy. Groups of 6 (treated) or 3 (control) infected hamsters were treated with ribavirin or PBS beginning on day 1 post-infection. On days 3, 5 and 7 post-infection therapy was terminated for one treatment and control group. (B) Effect of delayed ribavirin therapy. On days 3, 5 and 7 post-infection, a ten day therapeutic schedule consisting of daily ribavirin or PBS injections was initiated for one treatment or control group (6 and 3 infected hamsters, respectively).

Abbreviated ribavirin therapy at 50 mg kg^−1^ day^−1^ (a dose which in experiment one demonstrated similar levels of reduction in viral burden as therapy with 100 mg kg^−1^ day^−1^) administered by i.p. injection retained its protective efficacy against lethal ANDV infection in hamsters, even if only administered for three days, when treatment was initiated at day 1 p.i. (Figure 5A). Ribavirin therapy also remained 100% protective when treatments (50 mg kg^−1^ day^−1^) were initiated on day 3 p.i. (approximately 5 days prior to signs of disease in untreated hamsters); however the protective efficacy was drastically reduced when treatments were initiated on day 5 p.i. (survival rate 16.67%) and was completely ineffective when treatments began at day 7 p.i. (approximately 3 and 1 days prior to signs of disease in untreated hamsters respectively) (Figure 5B).

**Figure 6:**
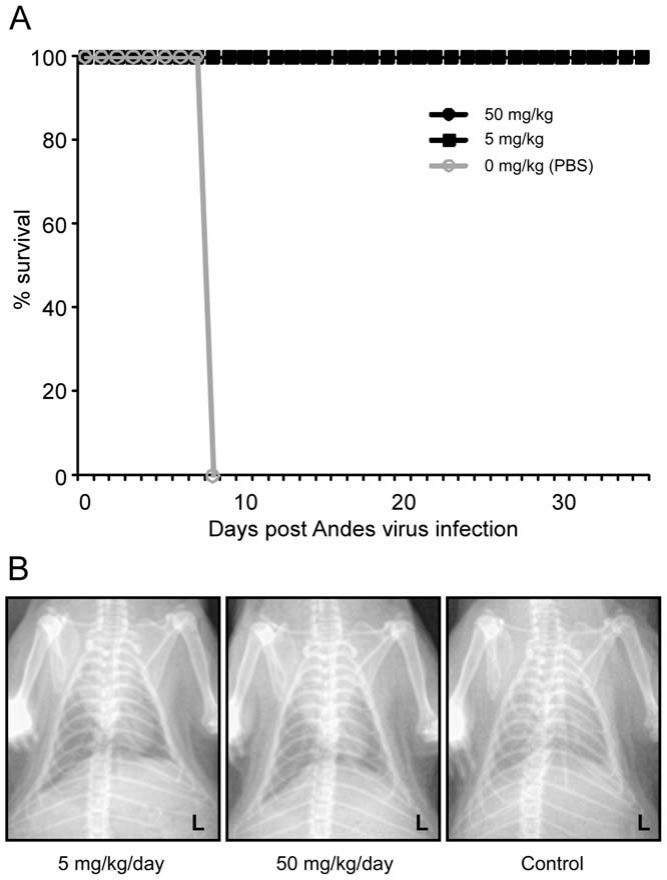
Protective efficacy of oral ribavirin therapy. (A) Effect of oral ribavirin therapy. Hamsters were infected with Andes virus as outlined in the material and methods section and treated with oral ribavirin at concentrations of 0, 5 or 50 mg kg^−1^ day^−1^ from day 1 through day 10 post-infection. (B) Chest radiographs of ribavirin treated and untreated hamsters. Extensive fulminant, bilateral lung infiltrates were observed at day 8 post-infection in control hamsters, whereas ribavirin treated hamsters showed little to no infiltrations. Shown are representative chest x-rays (ventral dorsal) from hamsters treated orally with 0, 5 or 50 mg ribavirin kg^−1^ day^−1^.

Similar to i.p. administration, daily oral treatment of ribavirin at both 5 and 50 mg kg^−1^ day^−1^ was 100% effective when administered from day 1 through day 10 p.i., though animals in the 5 mg treatment group appeared to display minor signs (lethargy and mild breathing abnormalities) of infection (Figure 6A). Chest x-rays performed on day 8 p.i. during the terminal phase of disease in control hamsters, revealed diffuse pulmonary infiltrates in control hamsters, which was absent in ribavirin treated animals (Figure 6B). The survival rate associated with oral ribavirin treatments at both concentrations was statistically significant (p = 0.0119).

Hantavirus nucleoprotein specific IgG antibodies were detected in convalescent serum samples collected from all surviving hamsters in all treatment groups at titers ≥6400 confirming ANDV infection. (data not shown).

## Discussion

The treatment and prevention of HPS has been a topic of intense interest since the initial description of the disease in 1993; however, as of yet no antivirals or specific vaccines exist to treat or prevent this disease [5], [15]. Currently the prevention of HPS relies solely on educational campaigns aimed at reducing contact with rodent hosts and their excreta/secreta, and treatment is mainly supportive care including intubation and mechanical ventilation as well as extracorporeal mechanical oxygenation in severe cases [2], [26]. To-date, few antivirals have been evaluated for efficacy against hantaviruses, and none have been tested in a lethal animal model of HPS.

*In vitro*, we found ribavirin to be a potent inhibitor of ANDV replication with an IC_50_ value between 5 and 12.5 µg ml^−1^ (Figure 1A), which is similar to that reported for SNV and Hantaan virus, and considerably lower than values reported for other viruses such as Rift Valley fever and yellow fever viruses [7], [16]. Importantly, the IC_50_ is similar to that of Lassa virus (16 µg ml^−1^) [27], suggesting that a therapeutic strategy similar to that used for Lassa fever would achieve plasma concentrations of ribavirin sufficient to be effective against ANDV [28]. Treatment resulted in significantly decreased viral RNA levels in infected cells and decreased production and release of viral particles, especially at ribavirin concentrations of 25 µg ml^−1^ or greater, which correlated with decreased nucleoprotein production and reduced viral yields (Figure 1A–D). *In vivo*, treatment with as little as 5 mg ribavirin kg^−1^ day^−1^ resulted in 100% protection from lethal disease in hamsters (Figure 2A), although some animals appeared to demonstrate mild physical signs of disease. Treatment with 25 mg kg^−1^ day^−1^ or greater completely abrogated both morbidity and mortality associated with HPS disease in hamsters, and resulted in significantly reduced viral loads as demonstrated by quantitative RT-PCR and IHC (Figures 2B–C and 3). Interestingly, while a dose-dependent reduction in viral RNA detection was observed in lung and liver samples from ribavirin treated hamsters, specimens from the same animals stained for hantaviral antigen by IHC were indistinguishable from one another upon blinded evaluation. The mechanism of antiviral activity for ribavirin is not completely understood and likely involves different methods depending on the target virus. Its mode of action has been associated with inhibition of the inosine monophosphate dehydrogenase (IMPDH) enzyme, direct inhibition of the viral RNA polymerase and lethal RNA mutagenesis [8]. Previously, it has been shown that ribavirin treatment increases the mutagenic frequency of Hantaan virus infection *in vitro*, leading to reduced antigen production despite detectable viral mRNA, and thus demonstrating an important role of lethal mutagenesis [10], [29]. Similar to these studies, our findings of reduced detection of viral antigen, despite considerable amounts of viral RNA (especially in the lowest treatment groups) lend support to the hypothesis of lethal mutagenesis as an important mechanism of ribavirin's antiviral activity against hantaviruses. Unfortunately, sufficient samples to thoroughly address this question were not collected as a part of these studies, and therefore will be the subject of future experiments.

As noted above, HPS disease progression in ribavirin treated animals was limited to mild physical signs (lethargy and minimal breathing abnormalities) in the 5 mg kg^−1^ day^−1^ treatment groups. Histological analysis of lung samples demonstrated no signs of edema or lung infiltration associated with HPS, which was supported by chest radiographs of a subset of treated and control hamsters (Figures 3 and 6B). Analysis of the host immune responses demonstrated considerable activation of pro- and anti-inflammatory Th1 (TNF α, IFN γ) and Th2 (IL-6, IL-10) cytokines in PBS treated hamsters, similar to that described in lethal cases of HPS in humans [30], which were largely absent in ribavirin treated hamsters, especially those receiving higher concentrations of the drug (Figure 4). Interestingly, unlike hantaviral antigen detection, the reductions in host responses occurred in a dose-dependent manner suggesting the requirement of ANDV viral replication in HPS disease progression.

The effectiveness of ribavirin therapy in hamsters was dependent on the duration of infection. When administered within three days after infection, ribavirin remained 100% effective; however when therapy was initiated at five days p.i., its efficacy dropped dramatically (Figure 5). This period correlates with the first detection of ANDV RNA in blood samples collected from untreated hamsters challenged i.p. with the same dose of virus (unpublished data); suggesting ribavirin monotherapy is only effective when initiated prior to, or at the time of, systemic spread of virus. When therapy was initiated at 7 days p.i. (i.e. within 24–72 hours of death in PBS treated hamsters), ribavirin had no effect on the disease outcome. Based on the immunopathology of HPS, these results are not surprising. If even possible, to achieve protection from lethal disease in the late stages of infection will most likely require a combination therapeutic approach involving a component to inhibit viral replication as well as a component(s) to modulate the deleterious host immune responses. Similar conclusions can be drawn from the findings of two clinical trials testing the efficacy of ribavirin monotherapy for HPS patients [17], [18]. The parallels between the results obtained here and those of the clinical trials assessing the efficacy of ribavirin against HPS further validate the hamster model of HPS and demonstrate its value in assessing therapies aimed at reducing morbidity and mortality associated with HPS.

The results of this study show the utility of ribavirin therapy for inhibiting ANDV replication and preventing lethal HPS disease in a highly sensitive animal model. As such, intravenous ribavirin should be considered (re-considered) as a therapeutic option for patients with early clinical symptoms of HPS or people with exposures to confirmed infected rodents such as bites. Effective treatment of hamsters with oral ribavirin even after infection makes prophylactic treatment with oral ribavirin an interesting option to prevent human-to-human transmission in case of ANDV infections. Oral treatment could also be considered for people in endemic areas with bite injuries from rodents with unconfirmed infection status or similar exposure levels as confirmed HPS cases. In addition, ribavirin would be a useful countermeasure against potential infection with HPS causing hantaviruses in laboratory or field-related incidents [31].

## Acknowledgments

The authors thank Dr. Joseph Prescott (Division of Intramural Research, National Institute of Allergy and Infectious Disease, National Institutes of Health, (DIR, NIAID, NIH)) for valuable suggestions and discussions regarding these studies, Dr. Rachel LaCasse and Trent Bushmaker (DIR, NIAID, NIH) for assistance in animal procedures, Rebecca Rosenke, Dan Long and Dr. Dana Scott (DIR, NIAID, NIH) for the histological analysis and Anita Mora (DIR, NIAID, NIH) for help with the art work.
